# Does Adjunctive Clindamycin Have a Role in *Staphylococcus aureus* Bacteremia? A Protocol for the Adjunctive Treatment Domain of the *Staphylococcus aureus* Network Adaptive Platform (SNAP) Randomized Controlled Trial

**DOI:** 10.1093/cid/ciae289

**Published:** 2024-05-27

**Authors:** Keerthi Anpalagan, Ravindra Dotel, Derek R MacFadden, Simon Smith, Lesley Voss, Neta Petersiel, Michael Marks, Julie Marsh, Robert K Mahar, Anna McGlothlin, Todd C Lee, Anna Goodman, Susan Morpeth, Joshua S Davis, Steven Y C Tong, Asha C Bowen, the Adjunctive Clindamycin Domain-Specific Working Group for the Staphylococcus aureus Network Adaptive Platform (SNAP) Trial Group, Keerthi Anpalagan, Ravindra Dotel, Derek R MacFadden, Simon Smith, Lesley Voss, Neta Petersiel, Michael Marks, Joshua S Davis, Asha C Bowen, Marc Bonten, Asha C Bowen, Nick Daneman, Sebastiaan J van Hal, George S Heriot, Roger J Lewis, David C Lye, Zoe McQuilten, David L Paterson, J Owen Robinson, Jason A Roberts, Matthew Scarborough, Steve A Webb, Lynda Whiteway, Genevieve Walls, Todd C Lee, Dafna Yahav, Marjolein Hensgens, Matthew P Cheng, Susan Morpeth, Steven Y C Tong, Joshua S Davis

**Affiliations:** Wesfarmers Centre of Vaccines and Infectious Diseases, Telethon Kids Institute, Perth, Western Australia, Australia; School of Medicine, University of Western Australia, Perth, Western Australia, Australia; Department of Infectious Diseases, Blacktown Hospital, Westmead, New South Wales, Australia; Centre for Infectious Diseases and Microbiology, Westmead Hospital, Westmead, New South Wales, Australia; Faculty of Medicine, University of Ottawa, Canada, The Ottawa Hospital Research Institute, Ottawa, Ontario, Canada; Department of Infectious Disease, Cairns Hospital, Cairns, Queensland, Australia; Starship's Children Health, Te Toka Tumai Auckland, New Zealand; Victorian Infectious Diseases Service, The Royal Melbourne Hospital, at the Peter Doherty Institute for Infection and Immunity, Melbourne, Victoria, Australia; Department of Infectious Diseases, The University of Melbourne at the Peter Doherty Institute for Infection and Immunity, Melbourne, Victoria, Australia; Clinical Research Department, London School of Hygiene & Tropical Medicine, London, United Kingdom; Hospital for Tropical Diseases, University College London Hospital, London, United Kingdom; Division of Infection and Immunity, University College London, London, United Kingdom; Wesfarmers Centre of Vaccines and Infectious Diseases, Telethon Kids Institute, Perth, Western Australia, Australia; Centre for Epidemiology and Biostatistics, Melbourne School of Population and Global Health, Faculty of Medicine, Dentistry and Health Sciences, University of Melbourne, Parkville, Victoria, Australia; Clinical Epidemiology and Biostatistics Unit, Murdoch Children's Research Institute, Parkville, Victoria, Australia; Berry Consultants, Austin, Texas, USA; Clinical Practice Assessment Unit and Division of Infectious Diseases, McGill University, Montreal, Quebec, Canada; Medical Research Council Clinical Trials Unit, Department of Infectious Diseases, University College London, Guy's and St Thomas’ Foundation NHS Trust, London, United Kingdom; Middlemore Hospital, Auckland, New Zealand; Global and Tropical Health, Menzies School of Health Research, Darwin, Northern Territory, Australia; The Immunology and Infectious Diseases Unit, John Hunter Hospital, University of Newcastle, Newcastle, New South Wales, Australia; School of Medicine and Public Health, University of Newcastle, Newcastle, New South Wales, Australia; Victorian Infectious Diseases Service, The Royal Melbourne Hospital, at the Peter Doherty Institute for Infection and Immunity, Melbourne, Victoria, Australia; Department of Infectious Diseases, The University of Melbourne at the Peter Doherty Institute for Infection and Immunity, Melbourne, Victoria, Australia; Wesfarmers Centre of Vaccines and Infectious Diseases, Telethon Kids Institute, Perth, Western Australia, Australia; School of Medicine, University of Western Australia, Perth, Western Australia, Australia; Global and Tropical Health, Menzies School of Health Research, Darwin, Northern Territory, Australia; Department of Infectious Diseases, Perth Children's Hospital, Perth, Western Australia, Australia

**Keywords:** *Staphylococcus aureus* bacteremia, clindamycin, randomized controlled trial, pediatrics, adults

## Abstract

**Background:**

The use of adjunctive antibiotics directed against exotoxin production in *Staphylococcus aureus* bacteremia (SAB) is widespread, and it is recommended in many guidelines, but this is based on limited evidence. Existing guidelines are based on the theoretical premise of toxin suppression, as many strains of *S. aureus* produce toxins such as leukocidins (eg, Panton-Valentine leukocidin, toxic shock syndrome toxin 1, exfoliative toxins, and various enterotoxins). Many clinicians therefore believe that limiting exotoxin production release by *S. aureus* could reduce its virulence and improve clinical outcomes. Clindamycin, a protein synthesis inhibitor antibiotic, is commonly used for this purpose. We report the domain-specific protocol, embedded in a large adaptive, platform trial, seeking to definitively answer this question.

**Methods and Analysis:**

The *Staphylococcus aureus* Network Adaptive Platform (SNAP) trial is a pragmatic, randomized, multicenter adaptive platform trial that aims to compare different SAB therapies, simultaneously, for 90-day mortality rates. The adjunctive treatment domain aims to test the effectiveness of adjunctive antibiotics, initially comparing clindamycin to no adjunctive antibiotic, but future adaptations may include other agents. Individuals will be randomized to receive either 5 days of adjunctive clindamycin (or lincomycin) or no adjunctive antibiotic therapy alongside standard-of-care antibiotics. Most participants with SAB (within 72 hours of index blood culture and with no contraindications) will be eligible to participate in this domain. Prespecified analyses are defined in the statistical appendix to the core protocol, and domain-specific secondary analyses will be adjusted for resistance to clindamycin, disease phenotype (complicated or uncomplicated SAB) and Panton-Valentine leukocidin–positive isolate.

## Teaser

This article reports the protocol for the adjunctive treatment domain of the *Staphylococcus aureus* Network Adaptive Platform (SNAP) trial. The adjunctive treatment domain aims to test the effectiveness of adjunctive clindamycin in patients with *Staphylococcus aureus* bacteremia.

*Staphylococcus aureus* bacteremia (SAB) is a common cause of sepsis and invasive infections across the life-course, from neonates to the elderly [1]. It is one of the leading causes of hospital- and community-acquired bloodstream infection, with a mortality rate of up to 30% by day 90 following onset in adults [1]. Multiple virulence factors, enzymes, and exotoxins of *S. aureus* contribute to its pathogenicity [2] including hemolysins, nucleases, proteases, lipases, hyaluronidase, and collagenase [3, 4]. Many strains may also produce leukocidins (eg, Panton-Valentine leukocidin [PVL]), toxic shock syndrome toxin 1, exfoliative toxins, and various enterotoxins [2–4]. Limiting the expression and release of these factors and exotoxins by *S. aureus* could theoretically reduce its virulence, improve clinical outcomes, and ultimately reduce the mortality associated with SAB [5, 6].

Lincosamides, such as clindamycin and lincomycin, function by inhibiting ribosomal protein synthesis, leading to a reduction in the production of multiple exotoxins [6]. Other protein synthesis inhibitor antibiotics that have the potential to inhibit protein production in *S. aureus* include macrolides, linezolid, aminoglycosides, and tetracyclines [7]. Currently, clindamycin is the most frequently used due to its low cost, consistent protein synthesis inhibitor activity [6] and other theoretical advantages include the lack of inoculum effect [8], activity during stationary phase [8], and the repression of penicillin-induced exotoxin production [9, 10].

Human studies involving clindamycin for exotoxin inhibition have shown mixed results but are limited by low-quality evidence arising from case reports, case series, or small clinical trials (Table 1). Several small case reports of patients with *S. aureus* necrotizing pneumonia who received clindamycin or linezolid reported a decrease in PVL in expectorated sputum [11, 12]. In addition, a review of 92 cases of *S. aureus* necrotizing pneumonia (80% of isolates were methicillin-resistant *S. aureus* [MRSA]) found that antibiotic therapy that included an antitoxin agent (clindamycin or linezolid) was associated with lower mortality rates (*P* = .007) [13]. A retrospective study of 269 patients with complex bacterial skin infections (predominantly MRSA) reported a similar length of stay in the adjunctive clindamycin group compared with the monotherapy group (3.7 ± 1.4 days vs 4.0 ± 2.0 days; 95% confidence interval, −0.32 [−.74 to.10]; *P* = .14) [15]. Interestingly, a retrospective review of 289 cases of *S. aureus* infections reported no significant difference in 30-day mortality rates among a small subset of patients receiving adjunctive lincosomide/linezolid therapy in PVL-positive compared with PVL-negative *S. aureus* infections (2.7% vs 5.3%, respectively; *P* = .53) [14].

**Table 1.** Human Studies Reporting Adjunctive Clindamycin as an Antitoxin Antibiotic for *Staphylococcus aureus* Infection

Authors (Year of Publication)	Study Design	Sample Size, No. of Patients (Age Category)	Type of *Staphylococcus aureus* Infection	Summary of Study and Primary End-Point Results^a^
Rouzic et al (2010) [11]	Case report	3 (2 Adults; 1 neonate)	*S. aureus* necrotizing pneumonia (2 MSSA and 1 MRSA case)	All 3 patients with severe necrotizing pneumonia survived. Treatment included various antibiotics (clindamycin, linezolid, and rifampicin); 2 patients also received IVIG. One patient each underwent pleural decortication and pleural fluid drainage, and 1 had decreased PVL in sputum with the start of antitoxin therapy.
Pasquier et al (2010) [12]	Case report	2 (Adults)	*S. aureus* pneumonia (MSSA)	Both patients with o severe *S. aureus* pneumonia received clindamycin or linezolid and IVIG, and both survived. PVL detection in expectorated sputum was decreased, but the report did not provide conclusive proof that the improvement and PVL decrease was due to antitoxin therapy.
Li et al (2011) [13]	Case series	93 (Adolescents and adults)	*S. aureus* necrotizing pneumonia (MRSA [80% of isolates] and MSSA)	Retrospective review of 93 cases of *S. aureus* necrotizing pneumonia found that antibiotic therapy including an antitoxin (clindamycin or linezolid) was associated with a lower mortality rate (*P* = .007).
Boan et al (2015) [14]	Case series	289 (Adults)	Skin and soft-tissue *S. aureus* isolates for PVL testing (MSSA and MRSA)	Retrospective review of 141 PVL-positive patients and 148 matched controls (with PVL-negative MSSA or MRSA isolates) reported no significant difference in 30-d mortality rate with adjunctive lincosamide/linezolid therapy in PVL-positive vs with PVL-negative *S. aureus* infections (2.7% vs 5.3%; *P* = .53).
Wargo et al (2015) [15]	Retrospective cohort study	269 (Adults)	ABSSSIs	Retrospective review of 269 patients with ABSSSIs (MRSA in 70% of positive infection site cultures) reported a decrease in LOS, (primary end point) in the adjunctive clindamycin group vs the monotherapy group (3.7 ± 1.4 d vs 4.0 ± 2.0 d; 95% CI, −0.32 [−.74 to .10]; *P* = .14). In patients presenting with an abscess, a significant decrease in LOS was reported for the clindamycin group (3.6 ± 1.5 vs 4.4 ± 2.3 d; 95% CI, −0.82 [−1.49 to −.15]; *P* = .02).
Campbell et al (2022) [16]	RCT	34 (Adults and children)	Severe *S. aureus* infections	Pilot RCT of 34 participants (11 children) reported the primary end point (no. of days alive and free of SIRS) was similar in standard vs adjunctive clindamycin therapy groups. For the secondary outcome, all-cause mortality rate at 90 d, there were no deaths (0/17 [0%]) in the clindamycin vs 4 (4/17 [24%]) in the standard therapy group.

Abbreviations: ABSSSIs, acute bacterial skin and skin-structure infections; CI, confidence interval; IVIG, intravenous immunoglobulin; LOS, length of stay; MRSA, methicillin-resistant *S. aureus*; MSSA, penicillin-resistant, methicillin-susceptible *S. aureus*; PVL, Panton-Valentine leukocidin; RCT, randomized controlled trial; SIRS, systemic inflammatory response syndrome.

^a^Primary end-point results are provided if available.

An Australian and New Zealand practice and attitudes survey in 2019 revealed that 93% of infectious diseases physicians had equipoise and willingness to randomize patients with SAB to receive adjunctive therapy with clindamycin or not [17]. CASSETTE, a recent open-label, pilot, randomized controlled trial (RCT) in adults and children with severe *S. aureus* infections, evaluated the efficacy of standard therapy alone versus standard therapy plus adjunctive clindamycin [16]. Thirty-four participants (23 adults and 11 children) were randomized to adjunctive clindamycin (10 mg/kg per dose up to 600 mg 4 times daily intravenously for adults and children or 10 mg/kg per dose up to 450 mg 3 times daily orally as an optional step-down for adults and children) for 7 days or no adjunctive therapy [16]. Although no difference was detected in the primary outcome of systemic inflammatory response syndrome–free days by day 14, the 90-day mortality rate was 0% (0 of 17 participants) in the adjunctive clindamycin group versus 24% (4 of 17) in the standard therapy group [16]. While the pilot RCT was underpowered to determine the effectiveness of clindamycin, it demonstrated feasibility and provides the rationale to conduct a larger and more robust trial.

The *Staphylococcus aureus* Network Adaptive Platform (SNAP) trial is a multisite adaptive platform trial that will simultaneously answer multiple clinical questions about SAB management [18]. The trial intends to enroll at least 7000 (6000 adults and 1000 children) participants, and it includes key groups who are often excluded from RCTs, such as pregnant participants [19].

Currently there are 3 domains within the SNAP trial, assessing the choice of backbone antibiotics, the role of an early oral switch strategy, and an adjunctive therapy domain, which at this time consists of clindamycin (or lincomycin) compared with no adjunctive therapy. A domain defines a set of mutually exclusive, competing interventions sharing a common clinical mode of action or clinical context of use. SNAP has a core (master) protocol [18] and domain-specific appendices [20] containing specific information relating to the study interventions within each domain. Subgroup-specific appendices contain information related to pediatrics, pregnancy, and people who inject drugs. Each participating site may elect to participate in ≥1 domain. In addition, silos represent the group of participants who are defined by the antibiotic susceptibility of their infecting isolate (eg, methicillin-susceptible *S. aureus* [MSSA] or penicillin-susceptible *S. aureus* [PSSA] and MRSA). The adjunctive domain in the SNAP trial provides an ideal opportunity to test the hypothesis that adjunctive clindamycin is effective in reducing mortality rates in SAB.

## METHODS AND ANALYSIS

### Randomization

At the time of writing, participants are randomized equally to interventions within each domain at platform entry using a web-based module. Participants’ allocation in each domain is revealed at the time that domain-specific eligibility criteria are confirmed.

### Blinding and Unblinding

Once participants are enrolled into the trial platform, those consented to this domain will be randomized to adjunctive therapy (clindamycin or lincomycin) or no adjunctive therapy. Participants, investigators, and site personnel will remain blinded to the allocation until the domain-specific eligibility criteria are satisfied. If the participant is eligible for the domain, the allocation will be revealed, and the investigator and participant will be unblinded. As with all current domains within SNAP, study drugs are open label. On a study-wide basis, investigators, site, and study personnel will remain blinded to aggregate domain outcomes until the SNAP Data Safety and Monitoring Committee recommends halting recruitment to the domain for noninferiority, superiority, or futility or if the maximum platform recruitment target is met.

### Interventions

Table 2 details the interventions and the recommended doses of clindamycin for adult and pediatric participants. Adjunctive clindamycin will be given for 5 days. The recommended clindamycin doses were 600 mg given 3 times daily intravenously in adults and 15 mg/kg per dose with a maximum dose of 600 mg 3 times daily intravenously in children. These were based on French and UK guidelines recommending up to 900 mg per dose [21, 22] (Infectious Diseases Society of America guidelines for MRSA did not recommend adjunctive clindamycin in 2011 [23], with updates currently in progress [24]) and a hollow-fiber model supporting 600 mg as an appropriate dose to inhibit exotoxin production [25, 26]. Clindamycin is the preferred agent, but if unavailable it can be replaced by lincomycin.

**Table 2.** Dosing Table for *Staphylococcus aureus* Network Adaptive Platform (SNAP) Adjunctive Domain (Clindamycin) Intervention

Adjunctive Treatment	Dose	Substitutions	Renal Impairment	Oral Alternative
None	…	Nil	…	…
Clindamycin	600 mg (10 mg/kg/dose in children), given intravenously every 8 h for 5 d^a^	Intravenous lincomycin (600 mg every 8 h)	No dosage adjustment	450 mg (10 mg/kg/dose in children), given orally every 8 h

^a^On platform days 1–5.

Administering clindamycin orally is allowed for sites and investigators who prefer oral dosing for reasons of cost, convenience, or antimicrobial stewardship. The oral dose is capped at 450 mg given 3 times daily (Table 2), as this is the maximum licensed dose in most regions and higher doses tend to have poor gastrointestinal tolerability [27]. Study investigators viewed a 5-day course as the shortest duration likely to have clinical impact while balanced against the possibility of adverse effects, particularly *Clostridioides difficile*–associated diarrhea. An initial short-course treatment strategy was also believed to balance the likelihood that any effect of adjunctive clindamycin will be achieved early in the SAB course, while potential gastrointestinal side effects were presumed to be less likely with fewer days of treatment.

### Population

All patients with SAB at participating sites are eligible to participate in the domain, within 72 hours of the collection of the index blood culture.

### Eligibility Criteria/Inclusion/Exclusion Criteria

Patients are eligible to participate in this domain if they have *S. aureus* cultured from blood regardless of susceptibility results for clindamycin, and emerging susceptibility results will not change this assignment. Phenotypic clindamycin susceptibility testing on all isolates will be performed centrally at the conclusion of the trial, and prespecified secondary analyses will be performed based on resistance classification. Tables 3 and 4 detail the platform and domain-level inclusion and exclusion criteria. *C. difficile*–associated diarrhea (any severity) is a key domain-level exclusion criterion (Table 4).

**Table 3.** Platform-Level Exclusion and Inclusion Criteria

Inclusion Criteria	Exclusion Criteria
*Staphylococcus aureus* complex grown from ≥1 blood culture Admitted to participating hospital at anticipated time of eligibility assessment (or, in patients who have died, if they were admitted to this site anytime from the time of blood culture collection until the time of eligibility assessment)	Time of anticipated platform entry is > 72 h after collection of the index blood culture Polymicrobial bacteremia, defined as >1 organism (at species level) in the index blood cultures, excluding organisms judged to be contaminants by the treating clinicians Currently treatment with a *systemic* antibacterial agent that cannot be ceased (except for allowed antibiotics listed in Table 1, those with limited absorption from the gastrointestinal tract or negligible antimicrobial activity against *S. aureus*) Known previous participation in SNAP Known positive blood culture for *S. aureus* (from the same silo: PSSA, MSSA, or MRSA) between 72 h and 180 d before the time of eligibility assessment Treating team deems that enrollment in the study is not in the patient’s best interest Treating clinician believes that death is imminent and inevitable Patient is for end-of-life care, and antibiotic treatment is considered inappropriate Age <18 y if pediatric recruitment is not approved at the recruiting site

Abbreviations: MRSA, methicillin-resistant *S. aureus*; MSSA, methicillin-susceptible *S. aureus*; PSSA, penicillin-susceptible *S. aureus*; SNAP, *Staphylococcus aureus* Network Adaptive Platform.

**Table 4.** Domain-Level Inclusion and Exclusion Criteria

Inclusion Criteria	Exclusion Criteria
Patients are eligible regardless of *Staphylococcus aureus* susceptibility testing results for clindamycin	Patients will be excluded from this domain if they have any of the following at the time of eligibility assessment: Previous type 1 hypersensitivity reaction to lincosamides Currently receipt of clindamycin (lincomycin) or linezolid that cannot be ceased or substituted for Necrotizing fasciitis Current CDAD (any severity) Current severe diarrhea from any cause (defined as grade ≥3^a^ or increase of ≥7 stools/d over baseline) Known CDAD in past 3 mo, or CDAD relapse (new clinical episode of diarrhea within 3 mo of a previous diagnosis of CDAD, and thought by the treating clinician to be attributable to *Clostridioides difficile*) in the past 12 mo At the time of domain eligibility assessment, >4 h has elapsed since platform entry Treating team deems that enrollment in this domain is not in the patient’s best interest

Abbreviation: CDAD, *Clostridioides difficile*–associated diarrhea.

^a^Grade ≥3 based on Common Terminology Criteria for Adverse Events (CTCAE) (version 5).

### Data Collection

Along with data collected in the core SNAP protocol, specific data collection points are required as part of the adjunctive treatment domain. Domain-specific data collection points include administration of clindamycin (or lincomycin) on the specified days if the participant is in the adjunctive treatment arm, blood culture at day 5, prespecified adverse outcomes up to day 14 and C-reactive protein levels on day 5. Table 5 details the domain-specific data collection points.

**Table 5.** Domain-Specific Schedule of Visits and Follow-up

Platform Day	1	2–4	5	14	Acute Discharge^a^
Administer clindamycin or lincomycin (if in clindamycin group)	X	X	X	…	…
Avoid clindamycin or lincomycin (if in nonclindamycin group)	X	X	X	X	X
CRP	…	…	X	…	…
SIRS criteria	…	…	X^b^	…	…
Creatinine^c^	…	…	X	X^d^	…

Abbreviations: CRP, C-reactive protein; SIRS, systemic inflammatory response syndrome.

^a^Acute discharge defined as the end of the acute index inpatient admission.

^b^Based on the white blood cell count obtained on day 5 ±1.

^c^If creatinine is measured at platform entry as part of the core protocol.

^d^Measuring serum creatinine on day 14 ± 3 is mandated only during the total index hospital stay. If the patient has been discharged, this could still be collected as part of routine follow-up if clinically indicated, but this is not mandated by the protocol.

### End Points

The primary end point for all domains within the SNAP trial is the all-cause mortality rate at day 90 after platform entry. *C. difficile* diarrhea, as determined by a clinical laboratory in the 90 days following platform entry for participants ≥2 years of age, is one of the 15 core secondary end points. The domain-specific secondary end points for the adjunctive treatment domain have been chosen to additionally determine the clinical impact and adverse event profile of adjunctive clindamycin (Table 6). A preplanned subgroup analysis based on phenotypic and genotypic clindamycin resistance markers will occur, to inform the uncertainty of whether clindamycin susceptibility is important for the proposed adjunctive activity of clindamycin in the treatment of SAB.

**Table 6.** Domain-Specific Secondary End Points for the Adjunctive Treatment Domain

Secondary End Points
Proportion of platform participants with all-cause diarrhea any time from domain reveal to platform d 14 or acute hospital discharge, whichever occurs first a. Defined as ≥3 loose stools per day, as reported by the patient, a treating nurse or doctor, or reported in medical records Change in CRP level from platform d 1 until d 5 (±1) a. CRP at d 1 means any blood CRP measurement obtained on platform d 1 or the calendar day before platform entry; if there is >1 measurement, the value recorded is that obtained closest to the time of platform entry. Proportion of platform participants with persistent bacteremia, defined as positive blood culture on platform d 5 ± 1; if blood culture at d 2 or 3 is negative, then d 5 blood culture will be assumed to be negative Proportion of platform participants meeting ≥2 SIRS criteria simultaneously on platform d 5 a. Abnormal body temperature (<36°C or >38°C) b. Tachypnea or mechanical ventilation (respirations >20/min in adults; age dependent in children) c. Tachycardia (heart rate >90/min in adults; age dependent in children) d. Abnormal leukocyte count (based on white blood cell count obtained on d 5 ± 1) Acute kidney injury (modified KDIGO stage 1; defined as an increase in serum creatinine of ≥26.5 μmol/L from platform entry [baseline] to platform d 5 *or* an increase in serum creatinine by ≥1.5 times the level at platform entry [baseline] within 14 d of platform entry) The KDIGO guidelines for acute kidney injury (AKI) define AKI as: • Increase in serum creatinine by 0.3mg/dL (= 26.5 mmol/L) or more within 48 hours OR • Increase in serum creatinine to 1.5 times baseline or more within the last 7 days OR

Abbreviations: CRP, C-reactive protein; KDIGO, Kidney Disease: Improving Global Outcomes; SIRS, systemic inflammatory response syndrome.

### Sample Size

SNAP uses a bayesian adaptive trial design without a fixed sample size [18]. For complex trials such as the SNAP trial, no analytical formulas exist, and computer simulations are therefore used to estimate the trial operating characteristics. Under a maximum anticipated sample size of 7000 participants (6000 adults and 1000 children) and a scenario of no differences between any interventions in all domains, the piecewise type I errors were all ≤7%. The power for superiority in the adjunctive antibiotic domain is 93% for an odds ratio (OR) of 0.75 and 77% for an OR of 0.8; under the baseline assumption of a 90-day mortality rate of 15% in the control group. These simulation-based estimates of the study power incorporate a range of plausible effect sizes, a clinically relevant treatment effect size (an OR of 0.8 translates to a reduction in absolute mortality rate from 15% to 12%), and feasibility for recruitment to this domain. The domain also has a 69% probability of meeting a futility trigger for the test of superiority if there is truly no difference between the clindamycin and no clindamycin arms. The report detailing the full set of simulated trial operating characteristics, under a range of plausible scenarios, is available as an online supplement to the published statistical appendix [28].

### Statistical Analysis

The primary objective for this domain is to determine whether adjunctive clindamycin is superior to no adjunctive treatment. The SNAP primary end point (90-day mortality rate) is binary and modeled using a Bernoulli distribution with a logistic link function, where the general linear function includes parameters for the effect of each intervention in each domain, silo, and subgroup, interdomain interactions between interventions, ineligibility for a domain, prognostic baseline factors, country nested in region, and (calendar) time epoch. Bayesian methods are used, with weakly informative priors, and permit complete information sharing (borrowing) between silos (MSSA/PSSA/MRSA) for the adjunctive therapy domain (further details available in the published statistical appendix) [28].

The superiority of any intervention versus the domain reference is defined for the adult subgroup based on the posterior probability of an OR of <1 for the primary end point (where an OR <1.0 indicates a decrease in mortality rate for an intervention compared with the reference). A domain-stopping decision will be recommended for superiority if, at a scheduled analysis, the posterior probability of superiority is >99%. A domain-stopping decision of futility will be declared if, at a scheduled analysis, the posterior probability of an OR <0.83 (ie, 1/1.2) for the primary end point is <1%. If the thresholds for the decision criteria are not met within the domain, at any scheduled analysis, then recruitment into the domain will continue.

Scheduled primary analyses will be performed when every cohort of 500 participants in the platform reaches the day 90 end point to evaluate the domain-specific decision criteria. When maximum platform recruitment is reached or domain-specific criteria are satisfied for superiority or futility, then Bayesian analysis and reporting of secondary core and adjunctive domain end points will occur [18]. In addition, prespecified secondary analyses of the primary end point (Table 7) will be performed for the adjunctive therapy domain. Continuous, time to event, and ordinal secondary end points will be modeled using appropriate statistical distributions, link functions, and the same general linear function as for the primary end point, is documented in the published statistical appendix [28].

**Table 7.** Prespecified Secondary Analyses for *Staphylococcus aureus* Network Adaptive Platform (SNAP) Adjunctive Domain (Clindamycin) Intervention

Prespecified Secondary Analyses on the Primary Estimand
1. No resistance, inducible resistance, or constitutive resistance to clindamycin, with no clindamycin resistance defined as an isolate that tests fully susceptible to clindamycin on standard antimicrobial susceptibility testing 2. Severe disease phenotype versus not (defined as ICU/HDU admission at the time of platform entry) 3. Isolate with Panton-Valentine leukocidin detected versus not detected

Abbreviations: HDU, high dependency unit; ICU, intensive care unit.

### Data Monitoring and Safety

SNAP is overseen by an independent data safety monitoring committee. This comparative effectiveness trial follows the guidelines of the International Conference on Harmonization Good Clinical Practice. All the treatments being studied are known to be safe and are approved by regulatory agencies for each SNAP site for treating *S. aureus* infections. The level of reporting of adverse events depends on the regulatory requirements in each region, but at a minimum, sites will report all serious adverse events related to the treatments being studied. Common adverse effects such as kidney or liver damage and diarrhea caused by *C. difficile* will be recorded as key secondary safety end points. The trial will also closely monitor critical data points for all participants and have a central safety team to assess all serious adverse reactions.

## DISCUSSION

In vitro data [29], guidelines [23, 30] and a single small pilot RCT [6] support a potential role for the addition of clindamycin alongside standard treatment of SAB. Despite this potential, previous trials of adjunctive antibiotics have shown that additional antibiotics result in an increased burden of adverse events without clinical benefit [31, 32]. In this context, where some guidelines recommend therapy in the absence of clinical evidence, it is essential to determine whether adjunctive clindamycin (or lincomycin) adds benefit or, as suggested by some prior trials, increases the likelihood of harm. The CASSETTE trial [16] was too small to answer this question definitively, but the reduced mortality rate in the participants who received adjunctive clindamycin supports equipoise for this question to be answered in the larger SNAP trial. Clinician surveys have similarly confirmed equipoise [17].

In the CASSETTE trial, investigators were aiming to enroll participants with severe SAB because the a priori hypothesis was that this was where adjunctive therapy would offer maximal benefit [16]. In the trial, 22 of 34 participants (64.7%) were admitted to the intensive care unit, confirming that a severe SAB phenotype was enrolled [16]. However, this trial was limited by the time it took to enroll participants (almost 2 years to enroll 34 participants at 6 sites), and it was unable to answer whether all patients with SAB should receive adjunctive clindamycin. CASSETTE demonstrated the difficulties in identifying and enrolling a cohort of severe SAB cohort within a realistic time scale. Meeting these criteria placed undue burden on the trial, slowed recruitment, and minimized generalizability. To increase the trial's efficiency and determine the potential benefit of adjunctive clindamycin in patients with severe and nonsevere disease phenotypes, SNAP will include all patients with SAB. A predefined secondary analysis in those with severe disease phenotype will be reported.

The SNAP trial provides the opportunity to inform best practice and clinical care worldwide in the use of adjunctive clindamycin (or lincomycin) for all SAB. The available sample size is substantially larger than in any clinical trial for SAB to date, with recruitment progressing on target, and the study design provides early opportunities to discover either benefit or harm, through iterative scheduled analyses after every 500 participants reach the primary outcome.

In contrast to the presumed benefits of adjunctive treatment with clindamycin, other in vitro models have suggested possible drug interactions that diminish the efficacy of the backbone β-lactam antibiotics for SAB treatment. In vitro data addressing drug interactions for the treatment of SAB suggest that clindamycin in combination with a cell wall–active antibacterial agent may result in diminished clearance efficacy of antibiotics against *S. aureus,* but there are no supporting in vivo or clinical trial data for this [29]. The SNAP trial provides the opportunity to answer this question definitively, with the additional benefit of including all susceptibility phenotypes of SAB in the same clinical trial, thus answering the question concerning PSSA, MSSA, and MRSA to inform clinicians caring for patients globally.

The choice of dose and duration of clindamycin for the adjunctive domain was clinically derived, using experience to balance possible adverse effects with optimal dosing, the shortest duration thought by clinicians to have an effect, and the available in vitro evidence and guidelines. Alternatives considered include the higher dose recommended in the UK and French guidelines (900 mg) [21, 22]; shorter duration of 3 days, per the hollow-fiber models [25, 26]; and longer duration of 7 days, per the CASSETTE trial [16]. Future studies within the SNAP adjunctive domain may be able to test these alternative approaches.

**Figure 1.**
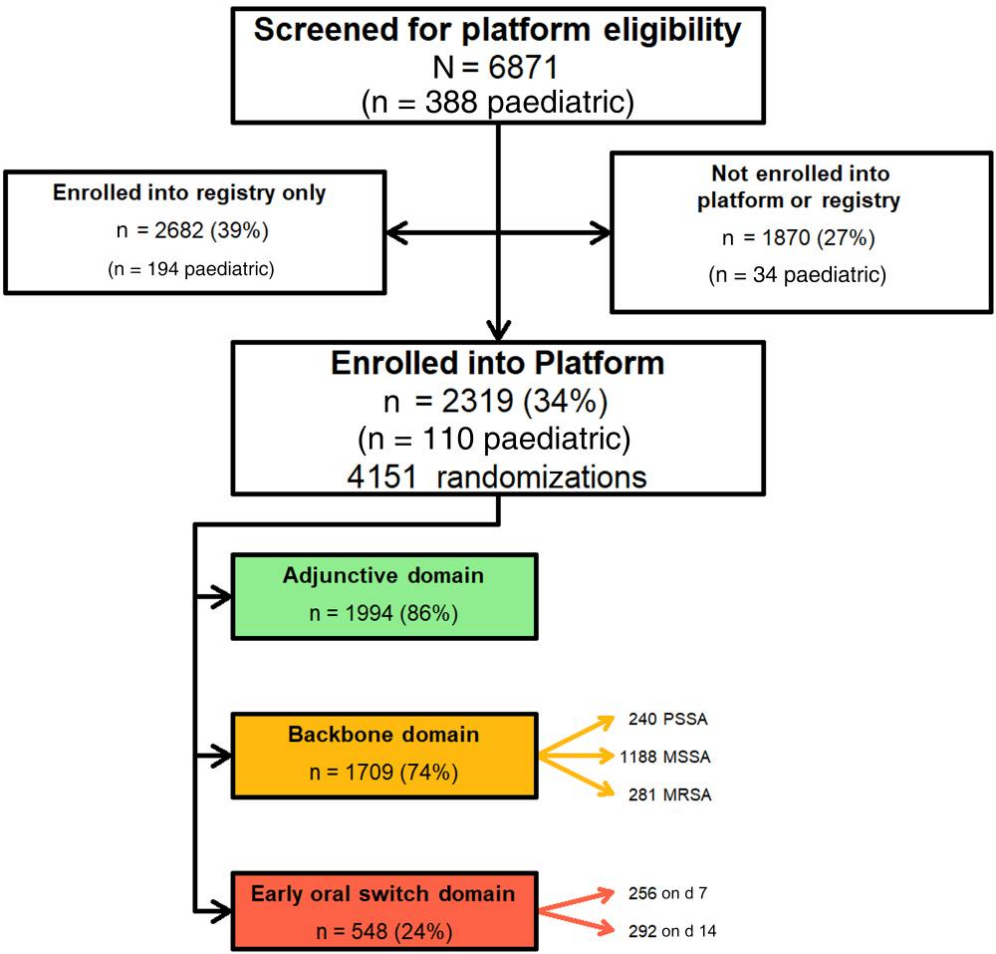
*Staphylococcus aureus* Network Adaptive Platform (SNAP) trial Consolidated Standards of Reporting Trials (CONSORT) diagram as of 26 March 2024. Abbreviations: MRSA, methicillin-resistant *S. aureus*; MSSA, penicillin-resistant, methicillin-susceptible *S. aureus*; PSSA, penicillin-susceptible *S. aureus*.

The SNAP trial started recruitment in February 2022 and has recruited 2319 platform participants as of 26 March 2024 (Figure 1). Of the 2319 platform participants, 1994 (86%) have been enrolled in the adjunctive clindamycin domain, making it the best recruiting domain within the SNAP trial. Despite strong recruitment and clinician equipoise, a minority of sites have elected not to participate in this domain due to concerns with increased risk of *C. difficile* toxin production, though some of these sites have gone on to activate this adjunctive domain.

Data from the adjunctive clindamycin domain of the SNAP trial are expected to be available in the coming years. Until then, clinicians should consider the available in vitro and limited in vivo evidence when considering the addition of clindamycin to standard treatment for SAB.

## Notes

***Author and study group contributions.*** The *Staphylococcus aureus* Network Adaptive Platform (SNAP) trial is an international platform with a large number of contributors. S. Y. C. T., and J. S. D. were instrumental in developing the original concept of the SNAP platform design and are senior SNAP platform leads. A. C. B. of the SNAP trial Adjunctive Clindamycin Domain-Specific Working Group conceived of and wrote the protocol for this part of the SNAP trial; thus, S. Y. C. T., J. S. D., and A. C. B. are joint lead senior authors for this article. K. A. wrote the first draft of the article and collated all further input and responses. All SNAP Adjunctive Clindamycin Domain-Specific Working Group authors—K. A., R. D., D. R. M., S. S., L. V., N. P., M. M., J. S. D., and A. C. B.—conceived of the idea and contributed to the design and final article preparation. Concepts, protocol design, and writing were improved, edited, and reviewed through the SNAP trial Global Trial Steering Committee (as composed at the time of drafting and writing), and its members are listed below. The SNAP study group members listed may differ from the groups listed in other manuscripts and are specific to the current article; these are collaborating authors who contributed to the design of the protocol and funding applications in their regions, though not directly to the writing of the manuscript.

***Acknowledgments.*** The authors thank the Data Safety and Monitoring Board (DSMC) and the Consumer Reference Groups and Public Patient Involvement groups in all regions for their input into protocol design.

***SNAP trial Global Trial Steering Committee.*** Steering committee members: Marc Bonten (UMC Utrecht, Utrecht University, Utrecht, the Netherlands); A. C. B.; Nick Daneman (Division of Infectious Diseases, Department of Medicine, Sunnybrook Health Sciences Centre, University of Toronto, Toronto, Canada); Sebastiaan J. van Hal (Department of Microbiology and Infectious Diseases Royal Prince Alfred Hospital, and School of Medicine, University of Sydney, Sydney, Australia); George S. Heriot (Department of Infectious Diseases University of Melbourne, Peter Doherty Institute for Infection and Immunity, Melbourne, Australia); Roger J. Lewis (Berry Consultants, Austin, Texas); A. G.; David C. Lye (National Center for Infectious Diseases and Tan Tock Seng Hospital, Yong Loo Lin School of Medicine, and Lee Kong Chian School of Medicine, Singapore); Zoe McQuilten (Monash University [including Australian and New Zealand Intensive Care Research Centre], Clayton, Australia, and Department of Haematology, Monash Health, Melbourne); David L. Paterson (University of Queensland Centre for Clinical Research, Royal Brisbane and Women's Hospital Campus, Brisbane, Australia); J. Owen Robinson (Department of Infectious Diseases, Royal Perth Hospital, and PathWest Laboratory Medicine, Perth; Department of Infectious Diseases, Fiona Stanley Hospital, and College of Science, Health, Engineering and Education, Murdoch University, Murdoch, Australia); Jason A. Roberts (University of Queensland Centre for Clinical Research, Royal Brisbane and Women's Hospital Campus, Herston Infectious Diseases Institute, Metro North Health, and Departments of Pharmacy and Intensive Care Medicine, Royal Brisbane and Women's Hospital, Brisbane; Division of Anesthesiology Critical Care Emergency and Pain Medicine, Niîmes University Hospital, University of Montpellier, Niîmes, France); Matthew Scarborough and Steve A. Webb (Department of Infectious Diseases, Oxford University Hospitals NHS Trust, Headington, Oxford, United Kingdom); Lynda Whiteway; Genevieve Walls (Middlemore Hospital, Auckland, New Zealand); T. C. L.; Dafna Yahav (Infectious Diseases Unit, Sheba Medical Center, Ramat-Gan, Israel); Marjolein Hensgens (UMC Utrecht, Utrecht University, and Julius Center for Health Sciences and Primary Care, University Medical Centre Utrecht, Utrecht); Matthew P. Cheng (Divisions of Infectious Diseases and Medical Microbiology, McGill University Health Centre, Montreal, Canada); S. M.; S. Y. C. T.; and J. S. D.

***Disclaimer.*** The views expressed are those of the authors and not necessarily those of the UK National Institute for Health and Care Research or the Department of Health and Social Care.

***Financial support*.** This work was supported by the Australian National Health and Medical Research Council (grants 2014900 and 1184238), the Australian National Health and Medical Research Council (grant 2017301), the Canadian Institutes of Health Research (grants 451092, 485083, and 451092), the Health Research Council of New Zealand (grant 20/344), the National Institute for Health and Care Research (grant 133719), the UK Medical Research Council (grant MC_UU_00004/05), the Singapore National Medical Research Council (grant CTGIIT21nov-0002), the National Institutes of Health (grant 1R01AI173138-01A1), the Netherlands Organisation for Health Research and Development (grant 10140022110014), and the Starship Foundation (New Zealand; grant ASF2144_WEBB).
